# Development of growth equations from longitudinal studies of body weight and height in the full term and preterm neonate: From birth to four years postnatal age

**DOI:** 10.1002/bdr2.1214

**Published:** 2018-03-14

**Authors:** John A. Troutman, Mary C. Sullivan, Gregory J. Carr, Jeffrey Fisher

**Affiliations:** ^1^ Central Product Safety, Mason Business Center, The Procter & Gamble Company Mason Ohio 45040; ^2^ University of Rhode Island, College of Nursing Providence Rhode Island 02903; ^3^ Data and Modeling Sciences, Mason Business Center, The Procter & Gamble Company Mason Ohio 45040; ^4^ National Center for Toxicological Research, Food & Drug Administration Jefferson Arkansas 72079

**Keywords:** growth, height, infant, longitudinal, neonate, pharmacokinetic, premature, weight

## Abstract

Physiologically based pharmacokinetic (PBPK) models are developed from compound‐independent information to describe important anatomical and physiological characteristics of an individual or population of interest. Modeling pediatric populations is challenging because of the rapid changes that occur during growth, particularly in the first few weeks and months after birth. Neonates who are born premature pose several unique challenges in PBPK model development. To provide appropriate descriptions for body weight (BW) and height (Ht) for age and appropriate incremental gains in PBPK models of the developing preterm and full term neonate, anthropometric measurements collected longitudinally from 1,063 preterm and 158 full term neonates were combined with 2,872 cross‐sectional measurements obtained from the NHANES 2007–2010 survey. Age‐specific polynomial growth equations for BW and Ht were created for male and female neonates with corresponding gestational birth ages of 25, 28, 31, 34, and 40 weeks. Model‐predicted weights at birth were within 20% of published fetal/neonatal reference standards. In comparison to full term neonates, postnatal gains in BW and Ht were slower in preterm subgroups, particularly in those born at earlier gestational ages. Catch up growth for BW in neonates born at 25, 28, 31, and 34 weeks gestational age was complete by 13, 8, 6, and 2 months of life (males) and by 10, 6, 5, and 2 months of life (females), respectively. The polynomial growth equations reported in this paper represent extrauterine growth in full term and preterm neonates and differ from the intrauterine growth standards that were developed for the healthy unborn fetus.

## 1 INTRODUCTION

Physiologically based pharmacokinetic (PBPK) models are being used increasingly by industry and regulatory agencies for quantitative understanding of the relationship between external exposures of xenobiotics and resulting internal concentrations of the parent and/or metabolites in blood or body tissues. The application of PBPK modeling in toxicology and risk assessment is particularly useful for evaluating potential pharmacokinetic differences that may occur between different routes of exposure, dose levels, species, and subpopulations. PBPK models are comprised of mathematical equations to represent physiology, in terms of organ volumes, tissue composition, and blood flows, and to describe the absorption, distribution, metabolism, and excretion of a chemical or drug in the body as a function of time. The incorporation of age‐appropriate biological descriptions into human PBPK models can be used in chemical risk assessment for neonates, infants, and children but the predictive accuracy and reliability of prospective model simulations is dependent on the ability of the model to describe accurately time‐dependent changes in physiology and other variables that influence a chemical's absorption and disposition. This includes body size, tissue composition, maturation of developing organs, such as kidney and liver, and the overall impact on renal and nonrenal elimination mechanisms.

Pharmacokinetic models and clinical data have been published for pediatric populations at various developmental stages providing insight into differences between neonates, infants, children, and adults (Abduljalil, Jamei, Rostami‐Hodjegan, & Johnson, 2014; Alcorn & McNamara, 2003; Clewell, Gentry, Covington, Sarangapani, & Teeguarden, 2004; Clewell et al., 2002; Edginton, 2011; Edginton, Schmitt, & Willman, 2006; Felter, Daston, Euling, Piersma, & Tassinari, 2015; Gentry, Covington, & Clewell, 2003; Ginsberg, Hattis, Miller, & Sonawane, 2004; Haddad, Restieri, & Krishnan, 2001; Lu & Rosenbaum, 2014; Maharaj & Edginton, 2014; Price, Haddad, & Krishnan, 2003; Yoon et al., 2011). The internal dose metric replaces the ingested or exposure dose to establish an internal dose‐response relationship that can then be used to assess potential differences in therapeutic efficacy or toxicity between the neonate, infant, and adult. The models must account for age‐specific differences in physiology, which include descriptions of developmental immaturity and growth. Chronological age, however, does not always correlate with functional age. Although there is an extensive body of literature reviewing the developmental differences between newborns, infants, and children, most of the information is based on subjects who have been born full term (≥37 weeks gestational age). Premature birth (<37 weeks) is a global problem and a leading cause of neonatal mortality (Liu et al., 2015). Each year, 15 million infants worldwide (World Health Organization, 2013) and 11% of all births in the United States (Martin, Hamilton, & Osterman, 2015) are born prematurely. Survivors of preterm birth are at risk of impaired physiological and biochemical functions associated with short‐ and long‐term disabilities and adverse outcomes including cardiovascular disease, type II diabetes, obesity, and lower cognitive performance (Blencowe et al., 2012; Kiserud et al., 2017; Liu et al., 2015). In the absence of accurate age‐ and time‐dependent descriptions of important physiological and biochemical changes in the preterm infant, the development and application of PBPK models in chemical risk assessment and drug development for this age group must rely on extrapolation methods to predict internal dose.

Postnatal growth and maturation of developing organs in preterm infants differ from those of full term infants. Although growth curves have been developed from descriptive anthropometric measurements or ultrasonographic data (Fenton & Kim, 2013; Kiserud et al., 2017; Usher & McLean, 1969), the growth charts that are currently available represent cross‐sectional or longitudinal intrauterine growth of healthy unborn fetuses or cross‐sectional data collected at birth or during fetal or neonatal autopsy (Archie, Collins, & Lebel, 2006; Phillips, Billson, & Forbes, 2009). Both approaches are useful for intrauterine growth monitoring and evaluating nutritional status of the developing fetus but they do not capture postnatal growth patterns of infants who are born preterm (Rao & Tompkins, 2007) and how an individual may change with postnatal age and maturation triggered by birth. Although longitudinal studies of growth in preterm infants have been published that include serial measurements of body weight (BW) and length/height (Ht) (Cole, Statnikov, Santhakumaran, Pan, & Modi, 2014; Guo, Roche, Chumlea, Casey, & Moore, 1997; Rochow et al., 2016), the data sets are limited by relative short durations of postnatal growth (e.g., birth to 42 weeks postmenstrual age, PMA) and are not in a form directly applicable for use in PBPK models. A recent publication by Claassen et al. (2015) described the development of a PBPK model for preterm neonates but descriptions of BW were obtained from published intrauterine growth charts. There is a need to develop accurate descriptions of postnatal longitudinal growth in subjects born preterm for the construction of PBPK models that are specific to preterm subpopulations from birth to early childhood and beyond.

The objective of this study was to assemble longitudinal BW and Ht data from subjects who were born full term or preterm and to develop mathematical equations describing postnatal growth in BW and Ht. These equations then can be used in PBPK models to describe age‐dependent changes in BW and Ht and maturation of physiological parameters that are calculated based on BW and Ht. Such models should be informative in developing predictive models for outcomes related to pharmacokinetics and the comparison of internal concentrations to chemical toxicity and drug efficacy in the preterm neonate as well as the impact of birth as a potential biological trigger for initiating maturation.

## 2 METHODS

### 2.1 Characteristics of published longitudinal data sets

Growth data were obtained from open‐source literature reporting longitudinal anthropometric measurements for BW and Ht for preterm (gestational age (GA)<37 weeks) or full term (GA ≥ 37 weeks) neonates (Table 1). Longitudinal data reported by Sullivan, McGrath, Hawes, and Lester (2008) (Table 2) consisted of 93 males and 100 females who were born in the United States at 24–42 completed weeks gestational age. The health status of each subject in this data set included groups of healthy full term (FT) and healthy preterm infants without major morbidities (HPT), a group of medical preterm infants with clinical illness but without neurological abnormality (MPT), a group of neurological illness preterm infants (NPT), and a group of small for gestational age preterm infants with or without medical problems (SGA) (Table 2). BW and Ht measurements at birth and 18, 30, and 48 months postnatal age were tabulated and individual subjects were stratified into one of five subgroups based on sex and gestational age at birth: 25 week GA subgroup (24–26 week GA), 28 week GA subgroup (27–29 week GA), 31 week GA subgroup (30–32 week GA), 34 week GA subgroup (33–36 week GA), and 40 week GA subgroup (37–42 week GA). This stratification was necessary because the growth trajectories were different depending on GA at birth. The five categories provided representative growth patterns for GA 25 to 40 weeks. For each subgroup and assessment age (i.e., birth, 18, 30, and 48 months), mean values for age, BW, and Ht were calculated from individual subject measurements. The initial polynomial equations that were developed exclusively using the Sullivan data set did not include weekly or monthly height and weight measurements needed to characterize rapid changes in growth between birth and 18 months of age.

**Table 1:** Details of data for deriving longitudinal growth equations

WHO classification	Extremely preterm(GA < 28 wks)		Very preterm(28 wks ≤ GA < 32 wks)				Moderate/late preterm(32 wks ≤ GA < 37 wks)		Term(GA ≥ 37 wks)	
Equation categories	25 wk GA subgroup(24–26 wk GA)		28 wk GA subgroup(27–29 wk GA)		31 wk GA subgroup(30–32 wk GA)		34 wk GA subgroup(33–36 wk GA)		40 wk GA subgroup(≥37 wk GA)	
	M	F	M	F	M	F	M	F	M	F
**Sullivan et al**. (2008)										
GA (wks) Mean (SD)	25.4 (0.843)	25.6 (0.535)	28.3 (0.802)	28.4 (0.770)	30.8 (0.907)	31.0 (0.775)	34.1 (1.03)	33.5 (0.855)	39.7 (0.644)	39.9 (1.12)
Birth wt (g) Mean (SD)	884 (15.2)	779 (9.54)	1158 (20.1)	1072 (26.6)	1481 (15.1)	1315 (20.1)	1545 (171)	1520 (272)	3421 (441)	3431 (381)
Length (cm) Mean (SD)	34.7 (2.46)	33.9 (2.13)	37.5 (3.76)	36.9 (3.01)	40.3 (2.34)	39.1 (3.00)	42.2 (1.89)	41.5 (3.23)	50.6 (2.81)	50.1 (1.73)
No. of subjects	10	7	25	24	22	31	15	14	21	24
Assessment period	Birth‐4 yrs	Birth‐4 yrs	Birth‐4 yrs	Birth‐4 yrs	Birth‐4 yrs	Birth‐4 yrs	Birth‐4 yrs	Birth‐4 yrs	Birth‐4 yrs	Birth‐4 yrs
**Ehrenkranz et al**. (1999)										
GA (wks) Mean (SD)	26.8 (2.0) (mixed‐sex)		28.5 (2.0) (mixed‐sex)		30.9 (1.9) (mixed‐sex)		–	–	–	–
%male	52.1		49.4		49.3		–	–	–	–
Birth wt interval (g)	801–900		1001–1200		1401–1500		–	–	–	–
No. of subjects	144		168		213		–	–	–	–
Assessment period	Birth‐73 days		Birth‐54 days		Birth‐28 days		–	–	–	–
**Bertino et al**. (2006)										
GA (wks) Mean (*SEM*)	–	–	29.8 (0.15) (mixed‐sex)		–	–	–	–	–	–
%male	–	–	not specified		–	–	–	–	–	–
Birth wt (g) Mean (*SEM*)	–	–	1225 (19)		–	–	–	–	–	–
No. of Subjects	–	–	94		–	–	–	–	–	–
Assessment period	–	–	2–24 months		–	–	–	–	–	–
**Casey et al**. (1990)										
GA (wks) Mean (SD)	–	–	–	–	–	–	35 (1.4)	35 (1.4)	–	–
Birth wt (g) Mean (SD)	–	–	–	–	–	–	2255 (138)	2255 (138)	–	–
Length (cm) Mean (SD)	–	–	–	–	–	–	45.3 (2.2)	45.3 (2.2)	–	–
No. of Subjects	–	–	–	–	–	–	114	99	–	–
Assessment period	–	–	–	–	–	–	Birth‐1 yr	Birth‐1 yr	–	–
**Cruise** (1973)										
GA range (wks)	–	–	–	–	–	–	33–36	33–36	37–42	37–42
Birth wt (g) Mean (SD)	–	–	–	–	–	–	2000 (300)	2000 (300)	3300 (400)	3300 (400)
Length (cm) Mean (SD)	–	–	–	–	–	–	43.6 (1.9)	43.6 (1.9)	50.1 (1.8)	49.5 (1.9)
No. of Subjects	–	–	–	–	–	–	40	43	60	53
Assessment period	–	–	–	–	–	–	Birth‐3 yr	Birth‐3 yr	Birth‐3 yr	Birth‐3 yr

**Table 2:** Characteristics and distribution of longitudinal data reported by Sullivan et al. (2008) from birth to 4 years of age

Neonatal		Number of	Number of	Average % of the population				
subgroup	Sex	subjects	measurements	FT	HPT	MPT	NPT	SGA
25 wk GA	males	10	34	0	0	53	38	8
	females	7	19	0	0	47	53	0
28 wk GA	males	25	89	0	3	45	47	5
	females	24	88	0	8	42	31	19
31 wk GA	males	22	66	0	33	49	18	0
	females	31	105	0	29	36	2	33
34 wk GA	males	15	50	0	23	18	8	50
	females	14	52	0	32	15	8	44
40 wk GA	males	21	66	100	0	0	0	0
	females	24	80	100	0	0	0	0
Total	males	93	305					
	females	100	344					
	M+F	193	649					

FT = full‐term; HPT = healthy preterm without morbidity; MPT = medical illness preterm; NPT = neurologic illness preterm; SGA = small for gestational age preterm.

Additional published literature with longitudinal BW and Ht measurements in newborns of varying ages were used to supplement each of the five GA subgroups that were initially developed from the Sullivan data set. Selected data sets from Ehrenkranz et al. (1999) included a total of 525 subjects (mixed‐sex) born in the United States with mean gestational ages of 26.8 weeks (144 subjects), 28.5 weeks (168 subjects), and 30.9 weeks (213 subjects) GA. Growth trajectories for BW and Ht were extracted from published curves using GetData Graph Digitizer (version 2.25.0.25) to obtain mean weight‐for‐age and mean height‐for‐age values that were reported from birth until a BW of 2000 g was reached. These data were subsequently incorporated into the 25, 28, and 31 week GA subgroups, respectively. Bertino et al. (2006) reported BW growth between 2 and 24 months from a population of 94 healthy (no major morbidities) very low birth weight preterms (29.8 week GA) collected from a single neonatal intensive care unit in Italy. Postnatal growth from this data set was extracted from published curves and incorporated into the 28 week GA subgroup. Mean BW and Ht data reported by Casey et al. (1990), collected from birth to 1 year of age, was incorporated into the 34 week GA subgroup and included 114 males and 213 females with a mean GA of 35 weeks. Cruise (1973) reported mean BW and Ht data from a total of 83 preterm infants (33–36 weeks GA) and 113 terms (37–42 weeks GA), from birth to 3 years of age, which were added to the 34 and 40 week GA subgroups, respectively. The postnatal BW growth pattern for the 34 week GA subgroup was characterized further by supplementing the measured data with BW predictions on postnatal days 7, 14, and 21 using a multiple regression model reported by Rochow et al. (2016). The use of this model was necessary because longitudinal data during the first few weeks of life was not available for describing the initial post birth weight loss and growth trajectory after completed postnatal adaptation. Anthropometric measurements reported in the NHANES 2007–2010 survey (Fryar, Gu, & Ogden, 2012), representing cross‐sectional growth in infants and children in the United States, were incorporated into the 40 week GA subgroup. The total number of measurements reported in the NHANES survey between birth and 4 years of age was 1,492 (males) and 1,378 (females) for BW and 1,452 (males), and 1,349 (females) for Ht.

### 2.2 Development of longitudinal growth equations for BW and Ht

To characterize growth trajectories in each GA subgroup, a series of polynomial equations were generated using Microsoft Excel to calculate BW and Ht as a function of postnatal days from birth to 4 years of age (Tables 3, 4, 5, 6). In most cases, it was necessary to develop multiple equations to characterize the rapid change in growth that occurred at different postnatal ages. In cases where multiple polynomials were developed, the fitting exercise was performed to ensure a smooth transition and proper alignment between adjacent regression equations. The *r*‐squared value for each polynomial was typically 0.995 or greater.

**Table 3:** Male body weight (grams) growth equations from birth to 4 years of postnatal age

Gestational age at birth (weeks)	Growth period	Equation	*R* ^2^
25	<73 days	1.650186 E –07*(days^6) – 3.972552 E –05*(days^5) + 3.685725 E –03*(days^4) – 1.667271 E –01*(days^3) + 4.012047E+00*(days^2) – 3.705484E+01*(days) + 8.887167E+02	0.9996
	73 days to 4 yrs	7.622771 E –06*(days^3) – 2.215779 E –02*(days^2) + 2.673944E+01*(days) + 1.526614E+02	1.000
28	<47 days	2.59724 E –06*(days^6) – 4.23233 E –04*(days^5) + 2.69872 E –02*(days^4) – 8.49142 E –01*(days^3) + 1.38614E+01*(days^2) – 9.52883E+01*(days) + 1.18552E+03	0.9967
	47 to 691 days	−2.62984 E –08*(days^4) + 7.17429 E –05*(days^3) – 7.68330 E –02*(days^2) + 4.37548E+01*(days) – 1.08330E+02	0.9998
	692 days to 4 yrs	5.884889 E –04*(days^2) + 3.571194E+00*(days) + 8.354553E+03	1.000
31	<30 days	1.374450 E –05*(days^6) – 1.525030 E –03*(days^5) + 6.816827 E –02*(days^4) – 1.566683E+00*(days^3) + 1.979696E+01*(days^2) – 1.113418E+02*(days) + 1.565761E+03	0.9996
	30 days to 4 yrs	−8.349630 E –09*(days^4) + 3.797827 E –05*(days^3) – 5.812052 E –02*(days^2) + 3.985814E+01*(days) + 7.754385E+02	1.000
34	<35 days	3.097386 E –03*(days^4) – 1.981930 E –01*(days^3) + 5.740936E+00*(days^2) – 4.577231E+01*(days) + 1.978709E+03	0.8967
	35 days to 4 yrs	−1.224875 E –08*(days^4) + 4.514750 E –05*(days^3) – 5.759340 E –02*(days^2) + 3.513539E+01*(days) + 2.432642E+03	0.9986
40	<4 yrs	1.076382 E –11*(days^5) – 5.095864 E –08*(days^4) + 9.191582 E –05*(days^3) – 7.825887 E –02*(days^2) + 3.746039E+01*(days) + 3.579470E+03	0.9829

**Table 4:** Female body weight (grams) growth equations from birth to 4 years of postnatal age

Gestational age at birth (weeks)	Growth period	Equation	*R* ^2^
25	<73 days	1.002907 E –07*(days^6) – 2.415770 E –05*(days^5) + 2.234812 E –03*(days^4) – 1.010066 E –01*(days^3) + 2.532719E+00*(days^2) – 2.215122E+01*(days) + 8.411017E+02	0.9994
	73 days to 4 yrs	3.419099 E –06*(days^3) – 1.550523 E –02*(days^2) + 2.472815E+01*(days) + 2.658614E+02	1.000
28	<47 days	1.44627 E –06*(days^6) – 2.41190 E –04*(days^5) + 1.58273 E –02*(days^4) – 5.16579 E –01*(days^3) + 8.93645E+00*(days^2) – 6.26752E+01*(days) + 1.11750E+03	0.9997
	47 to 691 days	−8.45563 E –09*(days^4) + 4.75747 E –05*(days^3) – 6.65276 E –02*(days^2) + 4.21990E+01*(days) – 4.43265E+01	0.9992
	692 days to 4 yrs	3.611897 E –03*(days^2) – 1.898985E+00*(days) + 1.071189E+04	0.9999
31	<30 days	−3.207719 E –05*(days^6) + 3.039153 E –03*(days^5) – 1.080554 E –01*(days^4) + 1.743312E+00*(days^3) – 1.118843E+01*(days^2) + 1.974714E+01*(days) + 1.382673E+03	0.9816
	30 days to 4 yrs	−2.066720 E –08*(days^4) + 6.651954 E –05*(days^3) – 7.437038 E –02*(days^2) + 4.078938E+01*(days) + 7.613636E+02	1.000
34	<35 days	2.465457 E –03*(days^4) – 1.807624 E –01*(days^3) + 5.953984E+00*(days^2) – 5.491700E+01*(days) + 1.958661E+03	0.8531
	35 days to 4 yrs	−1.589406 E –08*(days^4) + 5.431490 E –05*(days^3) – 6.410561 E –02*(days^2) + 3.626894E+01*(days) + 2.100630E+03	0.9906
40	<4 yrs	−1.262722 E –08*(days^4) + 4.254248 E –05*(days^3) – 5.008009 E –02*(days^2) + 3.086214E+01*(days) + 3.580496E+03	0.9898

**Table 5:** Male length/height (cm) growth equations from birth to 4 years of postnatal age

Gestational age at birth (weeks)	Growth period	Equation	*R* ^2^
25	<79 days	8.905395 E –04*(days^2) + 4.139884 E –02*(days) + 3.375000E+01	0.9920
	79 days to 4 yrs	3.205441 E –08*(days^3) – 1.047770 E –04*(days^2) + 1.287676 E –01*(days) + 3.305652E+01	1.000
28	<4 yrs	3.14905 E –08*(days^3) – 1.05086 E –04*(days^2) + 1.29879 E –01*(days) + 3.52325E+01	0.9991
31	<4 yrs	3.662092 E –08*(days^3) – 1.099299 E –04*(days^2) + 1.254900 E –01*(days) + 3.901344E+01	0.9997
34	<400 days	−3.501005 E –09*(days^4) + 3.405542 E –06*(days^3) – 1.218821 E –03*(days^2) + 2.462250 E –01*(days) + 4.342661E+01	0.9965
	400 days to 4 yrs	−1.782066 E –05*(days^2) + 5.296016 E –02*(days) + 5.690691E+01	0.9990
40	<4 yrs	2.873236 E –08*(days^3) – 8.491953 E –05*(days^2) + 9.965299 E –02*(days) + 5.173054E+01	0.9831

**Table 6:** Female length/height (cm) growth equations from birth to 4 years of postnatal age

Gestational age at birth (weeks)	Growth period	Equation	*R* ^2^
25	<79 days	7.419743 E –04*(days^2) + 5.619694 E –02*(days) + 3.343850E+01	0.9981
	79 days to 4 yrs	6.733384 E –08*(days^3) – 1.889907 E –04*(days^2) + 1.801133 E –01*(days) + 2.941724E+01	1.000
28	<4 yrs	3.61874 E –08*(days^3) – 1.08198 E –04*(days^2) + 1.25024 E –01*(days) + 3.53636E+01	0.9995
31	<4 yrs	3.727585 E –08*(days^3) – 1.089800 E –04*(days^2) + 1.215347 E –01*(days) + 3.891467E+01	1.000
34	<400 days	−3.401428 E –09*(days^4) + 3.318122 E –06*(days^3) – 1.173090 E –03*(days^2) + 2.320859 E –01*(days) + 4.316323E+01	0.9945
	400 days to 4 yrs	−1.204102 E –05*(days^2) + 4.651414 E –02*(days) + 5.713291E+01	0.9958
40	<4 yrs	1.777345 E –08*(days^3) – 6.086589 E –05*(days^2) + 8.644990 E –02*(days) + 5.148881E+01	0.9849

### 2.3 Reference population data

Intrauterine growth charts reported by Fenton and Kim (2013) and Kiserud et al. (2017) were used for comparing the extrauterine growth predictions from the polynomial growth equations based on postmenstrual age. Fenton and Kim (2013) developed cross‐sectional intrauterine/fetal growth charts from six large population surveys representing 3,986,456 births from Germany, United States, Italy, Australia, Scotland, and Canada. Kiserud et al. (2017) used a total of 7,924 repeated ultrasound measurements from 1,387 healthy women from ten countries in Africa, Asia, Europe, and South America to establish longitudinal reference standards for estimated fetal weight (EFW) and common ultrasound biometric measurements in low‐risk singleton pregnancies. In addition, cross‐sectional fetal‐neonatal autopsy reference standards developed by Archie et al. (2006) and Phillips et al. (2009) were used for comparing model‐predicted weights at various postmenstrual ages. Postnatal growth predictions from each of the five male and female GA subgroups were compared to the multicenter WHO Child Growth Standards (WHO, 2006) from birth to 4 years of age.

### 2.4 Simulations of BW and Ht

Polynomial growth equations for all GA subgroups were implemented into Berkeley Madonna software (version 8.3.18; University of California, Berkeley, CA). Simulations of growth were performed based on the selection of sex, GA subgroup and duration of the simulation run (e.g., birth to 48 months postnatal age).

### 2.5 Catch up growth assessment

Catch up growth was assessed based on z‐scores that were calculated between model predicted BW and target mean and standard deviation reference values reported in the World Health Organization Multicentre Growth Reference Study Group (WHO, 2006). z‐Scores were calculated at monthly intervals from birth to 2 years using Equation (1). Catch up growth was qualified as present if the z‐score was above a value of −2.

(1) $$z_{i}=\;(x_{i}-\mu )/\sigma$$

where *x_i_* is the model predicted value at time, *i*, and μ and σ correspond to the mean and standard deviation value of the reference population, respectively.

## 3 RESULTS

Published data sets that were used to generate polynomial growth equations are shown in Table 1. The compiled data sets include longitudinal measurements that were obtained from 1,063 preterm infants who were distributed among the four preterm subgroups as follows: 25 week GA (*n* = 161), 28 week GA (*n* = 311), 31 week GA (*n* = 266), and 34 week GA (*n* = 325). With the exception of the data set by Bertino et al. (2006), which reported BW data collected in Italy, all anthropometric measurements were obtained from subjects who were born in the United States. Data sets that were incorporated into the 40 week GA subgroup include longitudinal measurements from 158 subjects (Cruise, 1973; Sullivan et al., 2008) and 2,872 cross‐sectional measurements that were obtained from the NHANES 2007–2010 survey (Fryar et al., 2012).

Growth equations that were developed for BW and Ht predictions are shown in Tables 3, 4, 5, 6. The set of polynomial equations represent age‐specific growth of neonates who were born at 25, 28, 31, 34, and 40 weeks postmenstrual age. The developed growth equations correspond to each of the five neonatal categories that have been defined by WHO: extremely preterm (GA < 28 weeks), very preterm (28 weeks ≤GA < 32 weeks), moderate/late preterm (32 weeks ≤GA < 37 weeks), and term (GA ≥ 37 weeks).

As shown in Table 7, model‐predicted birth weights for males and females at 25, 28, 31, 34, and 40 gestational weeks showed good agreement (<20% difference) with the fetal‐neonatal weight standards that were developed from large international cross‐sectional (Fenton & Kim, 2013) and longitudinal (Kiserud et al., 2017) data sets. Birth weight predictions for the 25 week GA subgroup were slightly higher than the intrauterine weight standards for both male and female neonates (difference ranging from 7 to 17%) and predictions for the 28, 31, 34, and 40 week GA subgroups were at or below intrauterine weight standards (difference ranging from 1 to 18%). By comparison, the estimated fetal weights reported by Kiserud et al. (2017) were typically higher than those reported by Fenton and Kim (2013) but differences in estimated weights between these two data sets were <15%.

**Table 7:** Comparisons between model‐predicted birth weight (grams) for male and female neonates and intrauterine fetal/infant weight (grams) reference standards according to gestational week

	Model prediction		Fenton and Kim (2013)		Kiserud et al. (2017)	
GA (wk)	Male	Female	Male	Female	Male	Female
25	856	821	737	689	795	758
28	1,103	1,063	1,079	1,011	1,215	1,160
31	1,473	1,393	1,583	1,481	1,741	1,670
34	1,938	1,910	2,246	2,106	2,140	2,268
40	3,617	3,611	3,574	3,414	3,639	3,567

**Figure 1:**
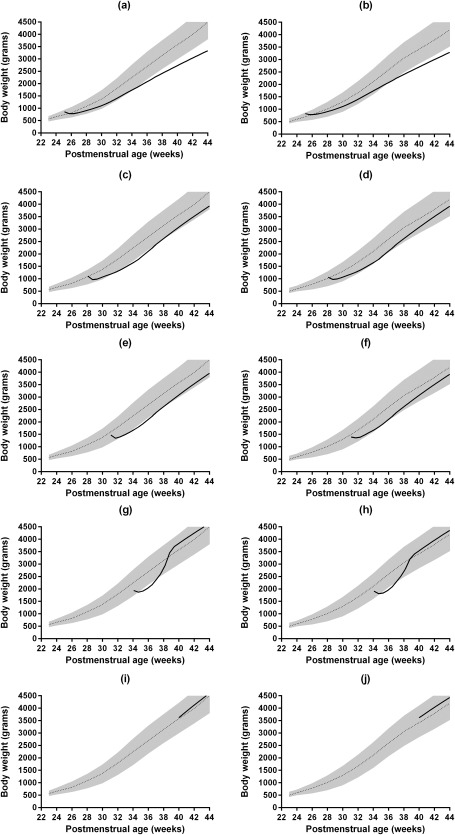
Comparisons of postnatal body weight growth predictions (solid line) in male (a, c, e, g, i) and female (b, d, f, h, j) neonates and the 10th, 50th, and 90th percentile (shaded gray area) cross‐sectional fetal/infant growth reference reported by Fenton and Kim (2013). 25 wk GA infants (a, b), 28 wk GA infants (c, d), 31 wk GA infants (e, f), 34 wk GA infants (g, h), 40 wk GA infants (i, j)

**Figure 2:**
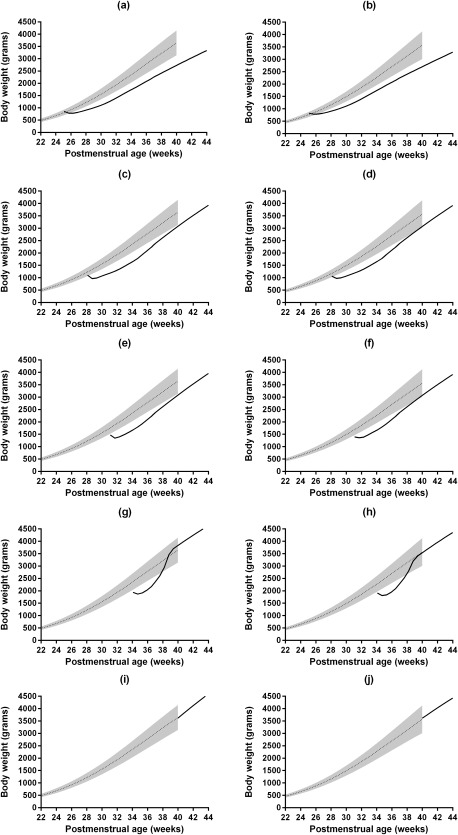
Comparisons of postnatal body weight growth predictions (solid line) in male (a, c, e, g, i) and female (b, d, f, h, j) neonates and the 10th, 50th, and 90th percentile (shaded gray area) longitudinal estimated fetal weight reference curve reported by Kiserud et al. (2017). 25 wk GA infants (a, b), 28 wk GA infants (c, d), 31 wk GA infants (e, f), 34 wk GA infants (g, h), 40 wk GA infants (i, j)

As shown in Figures 1 and 2, comparisons between model‐predicted extrauterine growth for preterm neonates and intrauterine growth for the developing fetus revealed slower growth for younger neonates. Preterm newborns lost weight during the first few days or weeks of life. After the postbirth weight loss, growth trajectories showed an increase in BW and the apparent recovery phase to achieve a term‐born growth pattern was longer in duration for neonates who were born at earlier postmenstrual ages. The BW growth trajectories for the 25 week GA male and female subgroups were at or below growth predictions for the 28, 31, 34, and 40 week GA subgroups for an extended time. The pattern of growth in males from the 28 and 31 week GA subgroups were consistent with the 10th percentile weight of babies reported by Fenton and Kim (2013) for intrauterine growth with similar weight values and incremental gains, while weight gains in females were between the 10th and 50th percentile. The duration of the recovery phase for the 34 week GA subgroup was shorter than recoveries from the 25, 28, and 31 week GA subgroups; model‐predicted BWs reached those of term‐born neonates between 38 and 40 weeks of postmenstrual age. The growth curve from the intrauterine reference standard reported by Kiserud et al. (2017) was consistently higher than postnatal BW predictions from the polynomial equations (Figure 2). Model‐predicted BWs for neonates who were born at 25, 28, and 31 weeks postmenstrual age were at or below the 10th percentile at 40 weeks of postmenstrual age.

**Figure 3:**
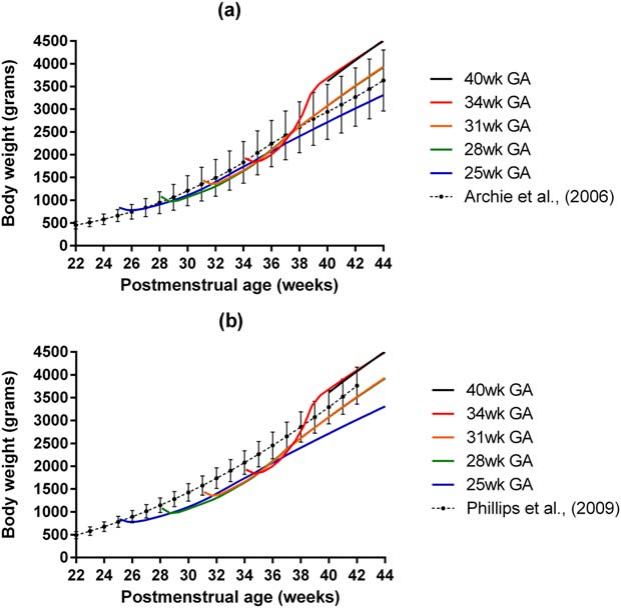
Averaged male and female postnatal body weight growth predictions from the 25, 28, 31, 34, and 40 week GA subgroup polynomial equations compared to mixed‐sex mean (st. dev.) body weight data that was collected during fetal or neonatal autopsy reported by Archie et al. (2006) (a) and Phillips et al. (2009) (b)

Figure 3 shows growth curves from the average of male and female BWs predicted by the model and mean mixed‐sex BW data that were measured during fetal/neonatal autopsy (Archie et al., 2006; Phillips et al., 2009). Postnatal growth predictions from the polynomial BW equations for the 25, 28, and 31 week GA preterm neonates were parallel to mean mixed‐sex BW reference standards. Body weights at 25, 28, 31, and 34 weeks of postmenstrual age were in very good agreement with corresponding mean autopsy values with differences <15% except for the 25 week PMA comparison to the Archie et al. (2006) data set (difference was 24%). The mean BW for the 40 week GA subgroup was higher than corresponding fetal‐neonatal autopsy values where the differences were 9 and 21%. Interestingly, the overall shapes of the curves for the mean (male and female) postnatal growth predictions in the preterm subgroups were more similar to the autopsy reference standards than the intrauterine growth standards for healthy unborn fetuses published by Fenton and Kim (2013) and Kiserud et al. (2017).

**Figure 4:**
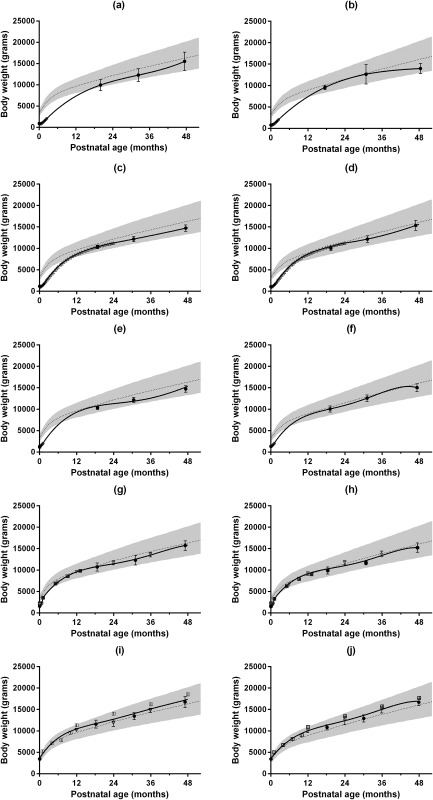
Growth trajectories for body weight in male (a, c, e, g, i) and female (b, d, f, h, j) neonates from birth to 48 months of postnatal age. 25 wk GA infants (a, b), 28 wk GA infants (c, d), 31 wk GA infants (e, f), 34 wk GA infants (g, h), 40 wk GA infants (i, j). Solid lines represent predicted body weights using the polynomial equations developed from compiled published longitudinal data sets. The gray shaded area represents the 5th, 50th, and 95th percentile body weights reported by the WHO Growth Standard (WHO, 2006). Red diamonds represent mean body weights reported by Ehrenkranz et al. (1999). Green triangles represent mean body weights reported by Bertino et al. (2006). Closed circles represent mean body weights reported by Sullivan et al. (2008). Orange inverted triangles represent mean body weights reported by Cruise (1973). Blue squares represent mean body weights reported by Casey et al. (1990). Brown circles represent mean body weights reported by Rochow et al. (2016). Open squares represent mean body weights reported by Fryar et al. (2012)

**Figure 5:**
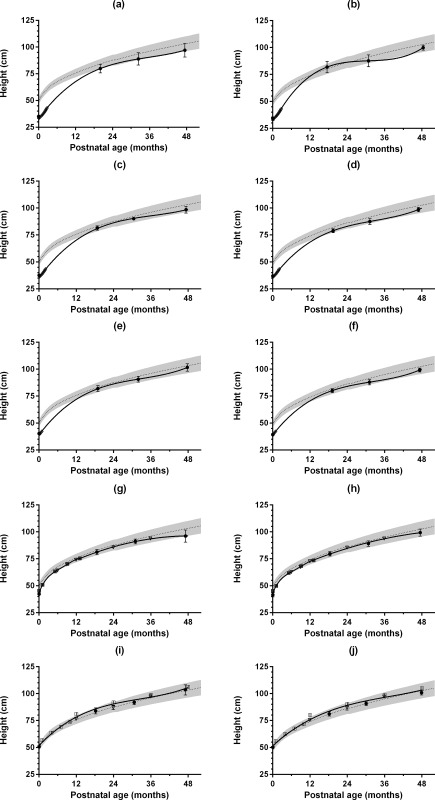
Growth trajectories for length/height in male (a, c, e, g, i) and female (b, d, f, h, j) neonates from birth to 48 months of postnatal age. 25 wk GA infants (a, b), 28 wk GA infants (c, d), 31 wk GA infants (e, f), 34 wk GA infants (g, h), 40 wk GA infants (i, j). Solid lines represent predicted body weights using the polynomial equations developed from compiled published longitudinal data sets. The gray shaded area represents the 5th, 50th, and 95th percentile body weights reported by the WHO Growth Standard (WHO, 2006). Red diamonds represent mean length/height reported by Ehrenkranz et al. (1999). Green triangles represent mean length/height reported by Bertino et al. (2006). Closed circles represent mean length/height reported by Sullivan et al. (2008). Orange inverted triangles represent mean length/height reported by Cruise (1973). Blue squares represent mean length/height reported by Casey et al. (1990). Brown circles represent mean length/height reported by Rochow et al. (2016). Open squares represent mean length/height reported by Fryar et al. (2012)

Figures 4 and 5 present model predicted growth trajectories for BW and Ht (solid black line) and measured data sets (symbols) that were used in the fitting exercise and the 5th, 50^th^, and 95th percentiles for term‐born infants (shaded area) reported by the WHO Growth Standard (WHO, 2006). As expected, model‐predicted changes in BW and Ht did not differ between the measured data sets that were used in the fitting exercise (Figures 4 and 5). The predicted BW and Ht values from model simulations were typically between the upper and lower confidence intervals of the published data. Birth weight predictions from the 25, 28, 31, 34, and 40 week GA subgroups were approximately 25%, 30%, 45%, 50%, and 110% of mean birth weights reported for male and female term neonates (WHO, 2006). In contrast, predictions of Ht at birth from preterm subgroups (i.e., 25, 28, 31, and 34 weeks GA) were closer to term values at birth, which ranged from 68% to 88% of mean birth heights reported for male and female full‐term neonates (WHO, 2006).

The BW and Ht growth curves from the 40 week GA subgroup was slightly higher than the 50th percentile values reported by WHO reference standard and typically within the 50th and 95th percentile values from birth to 4 years PNA. This finding might be explained by the fact that the data sets used to generate growth equations were obtained from subjects in the United States and tend to be greater in BW than those obtained from global survey data reported by WHO.

**Figure 6:**
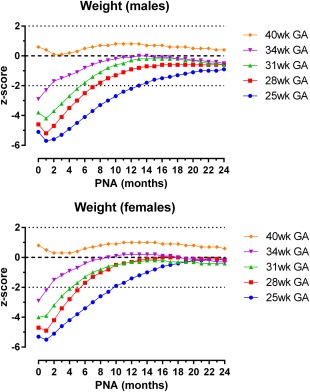
Body weight z‐scores for the 25, 28, 31, 34, and 40 week GA male and female neonate calculated from birth to 2 years postnatal age

Catch up growth for body weight at monthly intervals during the first 2 years of life was assessed by calculating the number of standard deviations between model‐predicted BW values and corresponding reference growth standards that were reported for subjects who were born full term (WHO, 2006). A z‐score of −2, which approximates the 2nd percentile, was used to determine whether catch up growth in BW was achieved. z‐Score values of −2 and 2 indicate that the predicted BW values are two standard deviations below and above the corresponding mean reference value, respectively. As shown in Figure 6, z‐scores at birth for all preterm subgroups were below −2 regardless of sex. Catch up growth for males born at 25, 28, 31, and 34 week GA was complete (i.e., z‐scores ≥ −2) by 13, 8, 6, and 2 months of life, respectively. In females, catch up in BW was complete by 10, 6, 5, and 2 months of life, respectively. In contrast, z‐scores for term‐born male and female 40 week GA subgroup ranged from 0.1 to 1.0, indicating a similar pattern of BW growth. This finding serves to verify the accuracy of the model predictions for the 40 week GA subgroup from birth to years of age, where BW predictions were within one standard deviation of the global reference standard reported by WHO (2006).

## 4 DISCUSSION

In 2010, the estimated preterm birth rate for 184 countries ranged from 5% in several northern European countries to 18% in Malawi, and an annual worldwide total of 14.9 million (11.1%) preterm babies (Blencowe, et al., 2012). The countries with the highest rates of preterm birth are in sub‐Saharan Africa and Asia (Blencowe et al., 2012). In the United States, 1 in 10 infants are delivered prematurely (Hamilton, Martin, Osterman, Curtin, & Mathews, 2015). Globally, preterm birth complications are the leading cause of death among children under 5 years of age (Liu et al., 2015). Babies who survive an early birth are more likely to experience short‐ and long‐term, adverse health and development outcomes than full term infants (Aarnoudse‐Moens, Weisglas‐Kuperus, van Goudoever, & Oosterlaan, 2009; Anderson, 2014; Bhutta, Cleves, Casey, Cradock, & Anand, 2002).

Rates of preterm birth and total preterm births increased or were stable in all but three of the 65 countries with reliable data and the average annual increase in preterm birth rate from 1990 to 2010 for developed regions, Latin America, and Caribbean was 1.1%, 0.5% and 1.5%, respectively (Blencowe et al., 2012). The decrease in neonatal mortality of neonates born preterm has led to a shift in neonatal care to improve the morbidity status associated with preterm birth and healthy development of survivors by healthcare practitioners. One of the issues regarding the body of evidence for premature infant development is the fact that most of the research for decades has focused on the very low birth weight infant (i.e., BW < 1,500 g), which account for less than 1.5% of the babies born in the United States. Approximately 84% of all preterm infants are born moderate/late (32 weeks < GA < 37 weeks) (Natarajan & Shankaran, 2016). Thus, there is a critical need to develop better tools to allow industry and regulators to address known data gaps in chemical and drug efficacy/safety assessments covering a wide spectrum of the growing preterm population.

We have compiled longitudinal growth measurements for BW and Ht from full term and preterm neonates from published literature. Polynomial equations were developed for calculating sex‐specific growth as a function of postnatal age, from birth to 4 years, for subjects who were born at mean gestational ages of 25, 28, 31, 34, and 40 weeks. The comparison of birth weight for each GA subgroup were within 20% of weight values from intrauterine reference standards (Fenton & Kim, 2013; Kiserud et al., 2017). The polynomial growth equations reported in this paper represent extrauterine growth in full term and preterm neonates and differ from the intrauterine growth standards that were developed for the healthy unborn fetus reported by Fenton and Kim (2013) and Kiserud et al. (2017). The intrauterine growth standards do not reflect actual growth of the preterm infant outside of the womb. After birth, the pattern of postnatal growth for preterm neonates reveal discrepancies between extrauterine growth (Figures 4 and 5) and intrauterine growth (Figures 1 and 2) where preterm neonates showed a relative loss in weight during the first few weeks of life, resulting in an offset in growth trajectories compared to intrauterine growth charts reported by Fenton and Kim (2013) and Kiserud et al. (2017). After the postbirth weight loss, postnatal growth trajectories showed a steady increase in BW. Weight gains in the 25 week GA male and female subgroups were consistently below the 10th percentile intrauterine weight at 40 week PMA. A comparison between the preterm growth predictions and the WHO reference standard (WHO, 2006) demonstrated slower gains in BW and Ht in subjects who were born at earlier gestational ages (Figures 4 and 5). Although not reflected by the model due to a lack of data, postbirth weight loss is expected for full term neonates.

Interestingly, age‐specific birth weights and patterns of growth from the preterm polynomial growth equations were generally consistent with the fetal‐neonatal autopsy reference standards shown in Figure 3 (Archie et al., 2006; Phillips et al., 2009). It is possible that this finding is a reflection of some underlying physiological condition or morbidity with the fetus or mother that resulted in an early birth prior to 37 weeks GA. The difference between preterm survivors and nonsurvivors might be attributed to substantial improvements in prenatal and postnatal care of infants with borderline viability by healthcare practitioners in recent years. Prior authors have shown that growth in premature infants who had neonatal illness can be delayed in the toddler and preschool ages, but seem to catch up with age (Sullivan, Msall, & Miller, 2012). Of course, the neonatal illness can be resolved, but ongoing or new health problems and nutrition can also affect growth. Although the effect of clinical illness on premature infant growth was not evaluated in this paper, it is reasonable to assume that the effect of health status on growth is represented in the model predictions since the longitudinal growth equations were derived from preterm subjects with and without major morbidities. Clinical illness is a significant factor for this subpopulation, which can potentially affect several important pharmacokinetic parameters.

The agreement between the model‐predicted birth weight and postnatal growth trajectories for the 40 week GA subgroup and published reference standards is particularly noteworthy. As shown in Figures 1 and 2 (*i* and *j*), body weight predictions from the model are aligned and in very good agreement with the 50th percentile intrauterine (Fenton & Kim, 2013; Kiserud et al., 2017) and cross‐sectional (WHO, 2006) reference standards for males and females at 40 weeks PMA. These findings serve as verification of the accuracy of the model in describing growth in the healthy full term population from birth to 4 years of age (Figure 4 and 5).

The postnatal longitudinal growth equations for BW and Ht that are presented in this manuscript can be used as the basis for developing mathematical descriptions of organ growth and maturation in subjects who were born between 25 and 40 weeks GA, from birth to 4 years of age. While the implementation of the growth equations into physiologically based pharmacokinetic models is not presented in the current paper, we expect BW and Ht to be an important data source for describing organ growth, similar to the model equations that have been reported by Bosgra, van Eijkeren, Bos, Zeilmaker, and Slob (2012). We are currently working to incorporate the longitudinal growth equations into a human physiological model to facilitate extrapolations between an external exposure and blood/tissue concentration‐time data for use in chemical risk and drug efficacy/safety assessments.

## CONFLICT OF INTEREST

None of the authors will benefit financially from this work other than their salaries. The authors declare that there are no conflicts of interest.

## DISCLAIMER

The views expressed in this paper are those of the authors and do not necessarily reflect the views of the US Food and Drug Administration.
